# Excess Mortality in California During the Coronavirus Disease 2019 Pandemic, March to August 2020

**DOI:** 10.1001/jamainternmed.2020.7578

**Published:** 2020-12-21

**Authors:** Yea-Hung Chen, M. Maria Glymour, Ralph Catalano, Alicia Fernandez, Tung Nguyen, Margot Kushel, Kirsten Bibbins-Domingo

**Affiliations:** 1 Institute for Global Health Sciences, University of California, San Francisco; 2 Department of Epidemiology and Biostatistics, University of California, San Francisco; 3 School of Public Health, University of California, Berkeley; 4 Department of Medicine, University of California, San Francisco

## Abstract

This time-series analysis examines the excess number of deaths across population subgroups in California during the COVID-19 pandemic.

Few studies on excess deaths during the coronavirus disease 2019 (COVID-19) pandemic in the US have documented how excess mortality varies across population subgroups.^1,2^ Using time-series models, we estimated excess deaths in California between March and August 2020 by age, sex, race/ethnicity, and educational level. California has a population of 39.5 million, which is approximately 12% of the US population of 328.2 million.

## Methods

Using California Department of Public Health data on deaths occurring on or after January 1, 2016, we estimated excess deaths during 2 COVID-19 pandemic periods: March 1 through May 9, 2020 (statewide shelter-in-place), and May 10 through August 22, 2020 (reopening). This study followed the Strengthening the Reporting of Observational Studies in Epidemiology (STROBE) reporting guideline. The study protocol was reviewed and approved by the institutional review board of the California Department of Public Health and the University of California, San Francisco.

We evaluated deaths for the entire state and for specific groups of interest defined by age, sex, race/ethnicity, and educational level. We restricted analyses of sex, race/ethnicity, and educational level to individuals aged 25 years or older. For each group of interest, we repeated the following procedure. We aggregated the data to weeks and fit dynamic harmonic regression models with autoregressive integrated moving average errors^3^ for the number of weekly all-cause deaths, using deaths occurring among group members between January 3, 2016, and February 29, 2020. Using the final model, we forecast the number of weekly deaths for each pandemic week. We estimated excess deaths for each week by subtracting the number of forecast deaths from the number of observed deaths. For each time period, we obtained 95% prediction intervals by simulating the forecast 10 000 times,^3^ selecting the 97.5% and 2.5% quantiles and subtracting the total number of observed deaths.

We obtained per capita estimates by dividing the excess deaths and corresponding 95% prediction intervals by population size, using estimates from the US Census Bureau.^4,5^ These data can be interpreted as risk differences; the exposure was the pandemic. We conducted all analyses in R, version 3.6.3 (R Project for Statistical Computing).

## Results

From March 1 through August 22, 2020, 146 557 deaths were recorded in California, with an estimated 19 806 (95% prediction interval, 16 364-23 210) deaths in excess of those predicted by historical trends (Table). Per capita excess mortality was highest among people aged 65 years and older, men, Black and Latino residents, and those without a college degree. Comparing deaths in March through April vs May through August, Latino residents and those without a high school degree or general education development (GED) certificate had the greatest increase in excess deaths, with Latino deaths tripling (from 16 to 51 excess deaths per million) and deaths in those without a high school degree/GED increasing by a factor of 3.4 (from 21 to 72 excess deaths per million). Across age groups, younger adults had the greatest increases in excess death, with rates more than doubling between shutdown and reopening (age, 25-54 years: from 4 to 11 excess deaths per million, 55-64 years: from 12 to 30 excess deaths per million).

**Table.** Excess Deaths Attributable to the COVID-19 Pandemic in California From March to August 2020, Using Time-Series Analysis of January 2016 to February 2020 Deaths

Variable	Excess deaths (95% PI)		Excess deaths per capita per week (95% PI)^a^		
	Total	Per capita^a^	March-April	May-August	Change^b^
Entire state	19 806 (16 364-23 210)	501 (414-587)	12 (8-16)	26 (21-30)	2.2
Age, y					
0-24	254 (53-453)	20 (4-36)	0 (0-1)	1 (0-2)	2.9
25-54	3377 (2987-3760)	207 (183-230)	4 (3-6)	11 (10-12)	2.7
55-64	2713 (2445-2980)	567 (511-623)	12 (8-16)	30 (27-33)	2.5
65-74	3564 (2947-4171)	1052 (870-1232)	24 (15-33)	54 (46-62)	2.2
75-84	4488 (3589-5377)	2638 (2109-3160)	71 (47-95)	128 (103-153)	1.8
≥85	5135 (3922-6307)	6849 (5230-8411)	171 (73-267)	342 (257-423)	2.0
Sex^c^					
Women	8182 (6913-9420)	596 (504-686)	13 (8-19)	31 (26-35)	2.3
Men	11 351 (9286-13 398)	859 (703-1014)	21 (14-28)	43 (36-51)	2.1
Race/ethnicity^c^					
Asian	2077 (1602-2546)	476 (367-583)	16 (11-22)	21 (16-26)	1.3
Black	1882 (1624-2135)	1206 (1041-1369)	40 (32-49)	54 (46-61)	1.3
Latino	8439 (7359-9493)	922 (804-1038)	16 (12-21)	51 (45-56)	3.1
White	5390 (3092-7632)	485 (278-687)	11 (1-20)	25 (15-34)	2.3
Educational level^c^					
No high school degree and no GED	5979 (5242-6705)	1300 (1140-1458)	21 (13-29)	72 (65-80)	3.4
High school degree or GED	6815 (5757-7857)	1230 (1039-1418)	32 (21-44)	60 (51-70)	1.9
Some college or associate degree	3242 (2091-4369)	413 (267-557)	10 (4-16)	21 (14-28)	2.1
Bachelor's degree or beyond	2606 (1989-3214)	291 (222-359)	8 (4-11)	14 (11-17)	1.8

Abbreviations: COVID-19, coronavirus disease 2019; GED, general educational development; PI, prediction interval.

^a^ Per 1 000 000 living individuals.

^b^ Multiplicative factor between first time period and second time period (calculated as ratio of second time period to first time period).

^c^ Among decedents aged 25 years or older.

In most weeks of the pandemic, Black residents had higher per capita excess mortality than other racial/ethnic group (Figure). Late in the shelter-in-place period, White, Asian, and Black residents had a decline in excess per capita mortality. In contrast, Latino residents and those without a high school degree/GED saw a substantial and sustained increase in per capita mortality.

**Figure.**
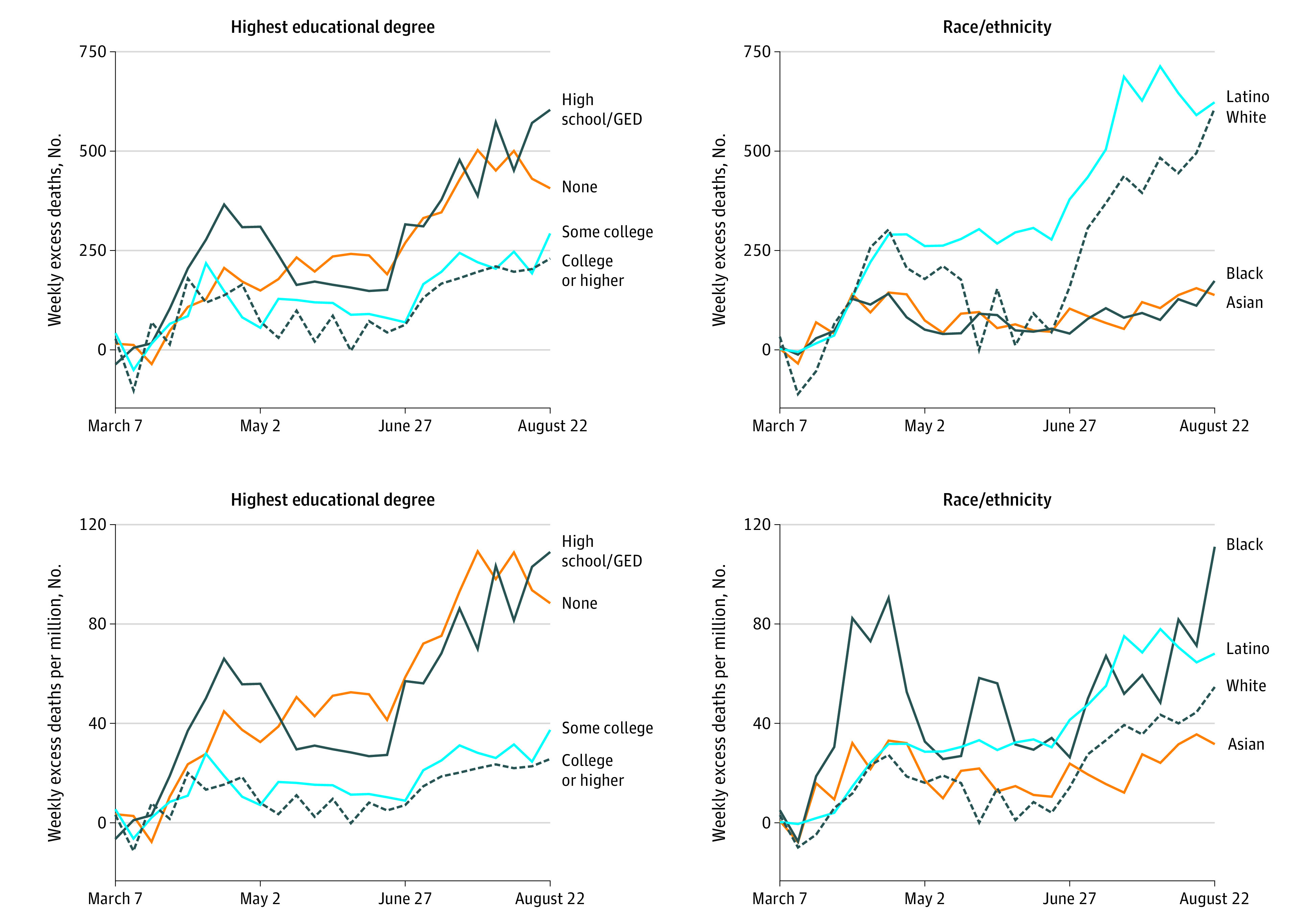
Excess Deaths During the Coronavirus Disease 2019 Pandemic In California From March to August 2020, Using Time-Series Analysis of January 2016 to February 2020 Deaths GED indicates general educational development.

## Discussion

During the COVID-19 pandemic in California, older adults, Black and Latino residents, and those without college degrees have experienced the highest per capita excess mortality. Following the statewide shelter-in-place, Latino residents and those without a high school degree/GED had the greatest increase in excess per capita mortality, with rates more than tripling after reopening. We hypothesize that this pattern reflects the risk of COVID-19 death faced by low-wage, essential workers and their social networks owing to occupational exposure, crowded housing, and inadequate access to testing or treatments.^6^

Although a limitation of this study is that our analyses were not designed to determine the associations with particular policies, our results suggest that the policies adopted to date have had disparate outcomes across population subgroups. Our findings underscore the importance of examining the inequitable effects of policies during the pandemic, reexamining the effects over time, and investing in strategies to mitigate the excess mortality in affected communities.

## References

[1] Woolf SH, Chapman DA, Sabo RT, Weinberger DM, Hill L, Taylor DDH. Excess deaths from COVID-19 and other causes, March-July 2020. JAMA. 2020;324(15):1562-1564. doi:10.1001/jama.2020.19545 33044483 PMC7576405

[2] Rossen LM, Branum AM, Ahmad FB, Sutton P, Anderson RN. Excess deaths associated with COVID-19, by age and race and ethnicity—United States, January 26-October 3, 2020. MMWR Morb Mortal Wkly Rep. 2020;69(42):1522-1527. doi:10.15585/mmwr.mm6942e2 33090978 PMC7583499

[3] Hyndman RJ, Athanasopoulos G. Forecasting: Principles and Practice. 2nd ed. OTexts; 2018.

[4] US Census Bureau. State population by characteristics: 2010-2019. Updated June 22, 2020. Accessed October 1, 2020. https://www.census.gov/data/tables/time-series/demo/popest/2010s-state-detail.html.

[5] US Census Bureau. 2018 Data profiles. Accessed October 1, 2020. https://www.census.gov/acs/www/data/data-tables-and-tools/data-profiles/.

[6] Michaels D, Wagner GR. Occupational Safety and Health Administration (OSHA) and worker safety during the COVID-19 pandemic. JAMA. 2020;324(14):1389-1390. doi:10.1001/jama.2020.16343 32936212

